# Stomach This: Autoimmune Atrophic Pangastritis, a Rare Type of Gastritis

**DOI:** 10.14309/crj.0000000000000549

**Published:** 2021-03-17

**Authors:** Sean Lee, Neha Varshney, Sasha Taleban

**Affiliations:** 1 Department of Medicine, University of Arizona College of Medicine, Tucson, AZ; 2 Department of Pathology, University of Arizona College of Medicine, Tucson, AZ; 3 Division of Gastroenterology and Hepatology, Department of Medicine, University of Arizona College of Medicine, Tucson, AZ

## Abstract

Atrophic gastritis can be environmental in origin and involve the antrum or autoimmune in origin and involve the body and fundus. We present a rare case of autoimmune atrophic pangastritis, a distinct type of autoimmune gastritis affecting the entire stomach, which should be considered in patients with other autoimmune disorders.

## INTRODUCTION

Atrophic gastritis is categorized as autoimmune (formerly type A) or environmental (formerly type B, most frequently from *Helicobacter pylori* infection), with the latter being the most common.^1^ These are classified by location, histology, and etiology. Autoimmune gastritis (AIG) inflames the stomach body and fundus and spares the antrum, whereas environmental gastritis inflames the antrum.^2^ Histologically, autoimmune atrophic pangastritis (AIAP) involves the autoimmune destruction of oxyntic mucosa and subsequent neuroendocrine (enterochromaffin-like cell) hyperplasia from the hypergastrinemia because of parietal cell loss.^3,4^ AIAP, a rare type of AIG, has been described in only 15 cases since being formally recognized in 2006.^1^ Risk factors include autoimmune and connective tissue disease, with women slightly more at risk. Clinical presentations vary widely, from nonspecific symptoms of nausea, vomiting, and abdominal pain to diarrhea, fat malabsorption, and malnutrition. Endoscopically, patients present across a spectrum from normal-appearing mucosa to multiple gastric ulcers.^5^ Therefore, diagnosis relies on histology and is characterized by intense mucosal lymphoplasmacytic inflammatory infiltrates, persisting even to severe glandular atrophy, pangastric distribution, lack of *H. pylori*, and lack of neuroendocrine hyperplasia.

## CASE REPORT

**Figure 1.**
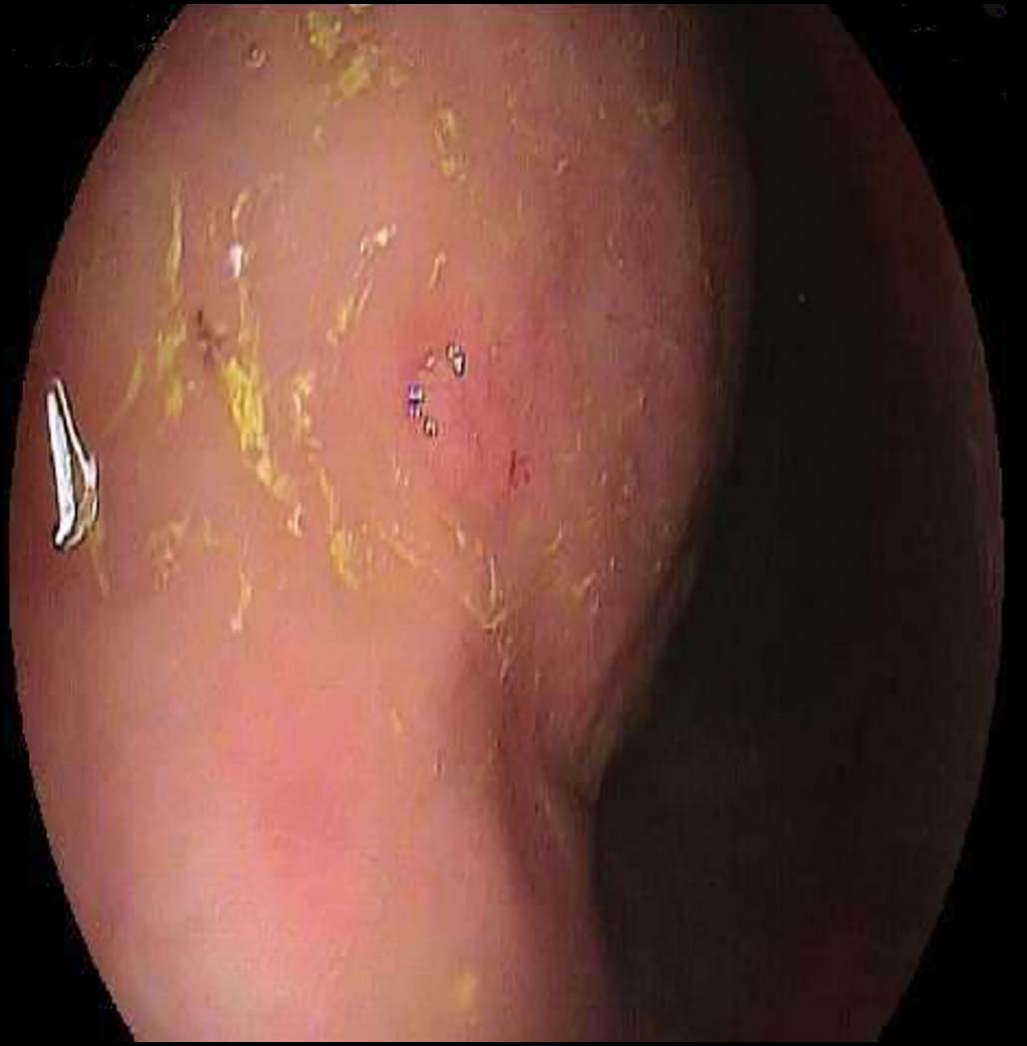
Endoscopy revealed erythema, congestion, friability, and diffuse atrophic mucosa throughout the stomach.

**Figure 2.**
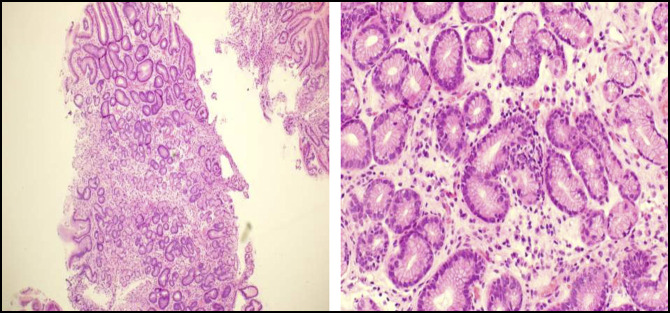
Gastric antrum biopsy: left: chronic inflammation. Right: chronic focal active gastritis (hematoxylin and eosin stain).

**Figure 3.**
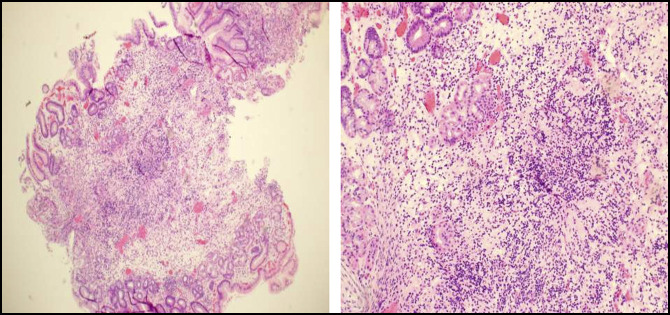
Gastric body biopsy: left: signs of chronic inflammation. Right: chronic inflammation and glandular dropout (hematoxylin and eosin stain).

**Figure 4.**
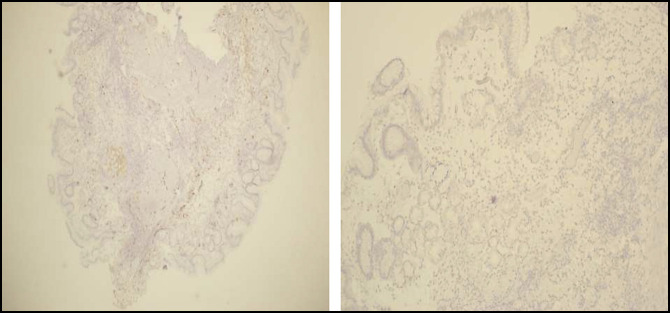
Gastric body biopsy: left: no *Helicobacter pylori* identified with immunohistochemical stain. Right: no neuroendocrine hyperplasia with synaptophysin stain (hematoxylin and eosin stain).

A 76-year-old woman with hypothyroidism (unknown cause), rosacea, and recurrent basal cell carcinoma presented in 2013 for further management of AIAP, which her private gastroenterologist initially diagnosed in 2012 after 2 months of persistent nausea and vomiting with a 10-pound weight loss. Endoscopy revealed erythema, congestion, and friability in the entire stomach (Figure 1). John's Hopkins University's pathologists confirmed initial biopsies by her private gastroenterologist. Biopsies from the antrum revealed chronic inflammation with chronic focal active gastritis (Figure 2). Gastric body biopsies also showed chronic inflammation with glandular dropout (Figure 3). No *H. pylori* or neuroendocrine hyperplasia was identified on multiple biopsies with hematoxylin and eosin staining and immunohistochemistry (Figure 4). Given a 95% sensitivity and 98% specificity for diagnosing *H. pylori* by histology, further testing for infection was not pursued. Laboratory work did not detect intrinsic factor antibodies.

The patient was started on prednisone and low-dose azathioprine (initiated after her basal cell carcinoma diagnosis), and responded with improved appetite and weight gain. Prednisone was tapered off, and she continued azathioprine monotherapy but held it from 2013 to 2014 because of recurrent basal cell carcinoma. She received numerous excisions (last one in 2017) for her basal cell carcinoma, including her cheek, arms, and back. She is currently in remission and continues to follow dermatology. She was later diagnosed with breast cancer in December 2015, underwent a right mastectomy in 2016, and completed chemotherapy and radiotherapy by November 2016. The patient's azathioprine was reduced during this time, and she complained of nausea and vomiting that is unclear if it was related to AIAP or from chemotherapy. Azathioprine was continued intermittently for 4 years total before weaning off because of long-standing clinical remission. The patient's AIAP remains in clinical remission off all therapy after 2 years. We plan to repeat an upper endoscopy this year.

## DISCUSSION

Our patient's atrophic gastritis with diffuse atrophy throughout the stomach, full-thickness mucosal inflammatory infiltrates not related to *H. pylori* infection, and lack of neuroendocrine hyperplasia are consistent with AIAP, a distinct form of AIG with unclear etiology. A case series of 8 patients showed a slight female predominance, systemic autoimmune and/or connective tissue disease in all, and autoimmune markers in 7 of 8 patients. Given its strong association with systemic autoimmune disease and the development of low-grade dysplasia in 1 patient, Jevremovic et al suggested an autoimmune etiology against multiple cell lineages with subsequent neoplastic potential. As much as 10% of AIG cases may progress to adenocarcinoma or carcinoid tumor. By contrast, the lack of neuroendocrine hyperplasia in AIAP suggests a lower risk of endocrine tumors, but the risk of adenocarcinoma persists.^5,6^

Autoimmune diseases such as lupus, type I diabetes, and vitiligo may all precede or follow the diagnosis of AIAP, similar to AIG.^7^ Interestingly, our patient was noted to have a history of hypothyroidism of unknown etiology that is currently well controlled on levothyroxine. Interestingly, up to 40% of those with Hashimoto thyroiditis have gastric disorders, and Hashimoto is present in about 40% of patients with autoimmune atrophic gastritis.^8^ Rosacea, present in our patient, may also have an autoimmune component and is associated with multiple immune-mediated gastrointestinal disorders such as celiac disease and inflammatory bowel disease.^9^ In patients with concomitant autoimmune diseases undergoing upper endoscopy, the finding of an autoimmune disorder should raise suspicion for the possibility of AIAP.

Our patient had a negative intrinsic factor antibody, but the use of traditional serum antibody testing for AIG is limited by low sensitivity and specificity.^5^ The ratio of pepsinogen 1 and 2, however, reflects the gastric mucosal morphologic state and may serve as a marker of pangastritis because pepsinogen 1 decreases as superficial gastritis progress to atrophy.^10,11^ The treatment of AIAP centers around immunosuppression. Given only a handful of case reports of AIAP, there are scant data regarding the best treatment modalities and clinical outcomes in adults. Most cases describe treatment with prednisone and/or azathioprine in pediatric patients. Symptoms most often resolve with treatment; however, on follow-up biopsies after treatment, abnormal findings of inflammation and dysplasia may persist for months, which support the risk for adenocarcinoma. Unlike AIG, there are no current guidelines for treating, screening, and monitoring patients with AIAP, and further investigation is warranted.^12–14^

## DISCLOSURES

Author contributions: S. Lee and S. Taleban wrote the manuscript and revised the manuscript for intellectual content. N. Varshney provided the pathology images and reviewed the literature. S. Taleban is the article guarantor.

Financial disclosure: None to report.

Informed consent was obtained for this case report.

## References

[1] Jacobson DL Gange SJ Rose NR Graham NM. Epidemiology and estimated population burden of selected autoimmune diseases in the United States. Clin Immunol Immunopathol. 1997;84(3):223–43. 9281381 10.1006/clin.1997.4412

[2] Harrison TR Braunwald E. Harrison's Manual of Medicine. 19th edn. McGraw-Hill Medical Pub. Division: New York, 2016, pp 1929–32.

[3] Pittman ME Voltaggio L Bhaijee F Robertson SA Montgomery EA. Autoimmune metaplastic atrophic gastritis: Recognizing precursor lesions for appropriate patient evaluation. Am J Surg Pathol. 2015;39(12):1611–20. 26291507 10.1097/PAS.0000000000000481

[4] Kohli Y Kato T Suzuki K Tada T Fujiki N. Incidence of atrophic gastritis with age in Japan and Canada. Jpn J Med. 1987;26(2):158–61. 3626154 10.2169/internalmedicine1962.26.158

[5] Jevremovic D Torbenson M Murray JA Burgart LJ Abraham SC. Atrophic autoimmune pangastritis: A distinctive form of antral and fundic gastritis associated with systemic autoimmune disease. Am J Surg Pathol. 2006;30(11):1412–9. 17063082 10.1097/01.pas.0000213337.25111.37

[6] Antico A. La gastrite autoimmune. RIMeL/IJLaM. 2008;4:125–33. [Italian.]

[7] Rodriguez-Castro KI Franceschi M Miraglia C. Autoimmune diseases in autoimmune atrophic gastritis. Acta Biomed. 2018;89(8-S):100–3. 30561426 10.23750/abm.v89i8-S.7919 PMC6502205

[8] Kalkan D Soykan I. Polyautoimmunity in AAG. Eur J Intern Med. 2016;31:79–83. 27085391 10.1016/j.ejim.2016.03.025

[9] Egeberg A Weinstock LB Thyssen EP Gislason GH Thyssen JP. Rosacea and gastrointestinal disorders: A population-based cohort study. Br J Dermatol. 2017;176(1):100–6. 27501017 10.1111/bjd.14930

[10] Massarrat S Haj-Sheykholeslami A. Increased serum pepsinogen II level as a marker of pangastritis and corpus-predominant gastritis in gastric cancer prevention. Arch Iran Med. 2016;19(2):137–40. 26838085

[11] Zayouna N. Atrophic Gastritis. Atrophic Gastritis. MedScape. (http://emedicine.medscape.com/article/176036-overview). Updated Dec 20, 2018. Accessed Jun 21, 2020.

[12] Kulnigg-Dabsch S. Autoimmune gastritis. Wien Med Wochenschr. 2016;166(13):424–30. 27671008 10.1007/s10354-016-0515-5 PMC5065578

[13] Park JY Lam-Himlin D Vemulapalli R. Review of autoimmune metaplastic atrophic gastritis. Gastrointest Endosc. 2013;77(2):284–92. 23199649 10.1016/j.gie.2012.09.033

[14] Neumann WL Coss E Rugge M Genta RM. Autoimmune atrophic gastritis—Pathogenesis, pathology and management. Nat Rev Gastroenterol Hepatol. 2013;10(9):529–41. 23774773 10.1038/nrgastro.2013.101

